# Increasing prosocial behavior and decreasing selfishness in the lab and everyday life

**DOI:** 10.1038/s41598-020-78251-z

**Published:** 2020-12-04

**Authors:** Andrew T. Gloster, Marcia T. B. Rinner, Andrea H. Meyer

**Affiliations:** 1 Division of Clinical Psychology and Intervention Science, Faculty of Psychology, University of Basel, Missionsstrasse 62 A, 4055 Basel, Switzerland; 2 Division of Clinical Psychology & Epidemiology, Faculty of Psychology, University of Basel, Basel, Switzerland

**Keywords:** Human behaviour, Social evolution

## Abstract

The tension between selfishness and prosocial behavior is crucial to understanding many social interactions and conflicts. Currently little is known how to promote prosocial behaviors, especially in naturally occurring relationships outside the laboratory. We examined whether a psychological micro-intervention would promote prosocial behaviors in couples. Across two studies, we randomized dyads of couples to a micro-intervention (15 min), which increased prosocial behaviors by 28% and decreased selfish behaviors by 35% a week later in behavioral games in a dose–response manner. Using event sampling methodology, we further observed an increase in prosocial behaviors across one week that was most pronounced in participants who received the intervention. These results from the laboratory and everyday life are important for researchers interested in prosocial behavior and selfishness and have practical relevance for group interactions.

## Introduction

Humans are capable of prosocial and cooperative behaviors to a degree that is unique among animals. This capacity is believed to have substantially contributed to human’s evolutionary advantage^1–3^. Humans are also capable of tremendous selfishness, self-interest, and cruelty. The tension between our prosocial and selfish tendencies is inherent in many laws and is crucial to understanding social interactions and societal conflicts.

Given its centrality, the tension between prosocial and more selfish behaviors has been discussed from numerous viewpoints. Different theoretical and methodological assumptions lead to different operationalizations of prosocial and selfish behaviors. Within the laboratory, one of the most widely utilized operationalizations are behavioral games. These place participants (almost always strangers) in standardized situations that force a choice between prosocial and selfish options. By standardizing this tension, behavioral games facilitate comparisons across studies. Despite their clarity and wide-spread utilization, behavioral games have been criticized for their lack of external validity. That is, the conditions standardized in the laboratory seldom, if ever, apply to everyday life^4^. For example, people do not usually enter into financial decisions with complete strangers they just met—conditions often utilized in such studies. Needed are studies that capitalize on laboratory studies’ advantages, while linking them to naturally occurring prosocial and selfish behaviors in everyday life and in naturally occurring groups, such as couples.

Towards the goal of better understanding prosocial behaviors in and out of the laboratory, it is important to target and test conditions and mechanisms that give rise to prosocial vs. selfish behaviors. One way to accomplish this is to experimentally intervene to promote prosocial behaviors in some participants and not others, thereby testing hypothesized mechanisms and theories of change^5^. To date, however, relatively few studies have actively intervened in an attempt to purposely promote skills that increase prosocial behaviors. Instead, research has concentrated on fixed factors (e.g., closeness, attraction, personality traits, etc. of the social partner^4,6^), game characteristics (e.g., payoff patterns^7^), and state factors largely outside the direct control of participants (e.g., negative mood, priming, etc.^8^). Some biological interventions have been tried^9^, but these too are outside the voluntary control of people in everyday situations. From both a scientific and public health perspective, it is important to develop interventions that individuals can willingly employ and then test to see to what degree they impact prosocial behaviors in the lab as well as everyday life and in naturally occurring groups with real-life long-term relationships.

Psychological flexibility, a set of malleable inter- and intra-personal skills that allows one to be “aware, open, and committed to behaviors that are congruent with deeply held values”^10,11^ is such a candidate intervention. A psychologically flexible person is clear about his/her authentic values, pursues actions in service of these values, and is even willing to do so when it means they may have uncomfortable negative experiences. Training psychological flexibility involves clarifying and specifying one’s chosen values (as opposed to externally driven goals). So defined, values are deeply and intrinsically important to people and their authentic self-chosen values almost always involve social relations^12^ (e.g., “I care about being a supportive spouse and being attentive to the needs of my family”). When training psychological flexibility, people also become aware how internal (e.g., thoughts, emotions, etc.) and external (e.g., interactions with other people, environmental constraints) aspects of life may get in the way or compromise how one wishes to act. Finally, psychological flexibility training helps people to deal differently with the perceived barriers (e.g., by accepting the thoughts and feelings) so that they can flexibly choose how they act (vs. rigidly react to and avoiding the perceived barriers). Psychological flexibility is considered a fundamental aspect of health and well-being, and is related to social interactions, including helping people actually follow through with socially related values^13–16^. As such, psychological flexibility is a promising target to test in its ability to increase prosocial behaviors.

Another way of targeting mechanisms is to frequently assess the variable of interest—here prosocial behaviors—often enough to capture its occurrence in its naturally occurring environments, situations, and routines such as in Event Sampling Methodology (ESM^17^). ESM captures participants experiences in real time, often with the use of technology, which has the advantage of maximizing ecological validity, reducing recall bias, and allowing for the examination of potential mechanisms in everyday contexts^18,19^. Thus, combining laboratory and ESM capitalizes on the advantages of each method while balancing out some of their limitations.

With the goal of linking laboratory and field studies, we examined whether a micro-intervention promoting psychological flexibility could increase prosocial behavior in naturally occurring relationships. Partners or couples are the most prevalent naturally-occurring social group and thus an important test of the generalizability of behavioral games that are usually tested in strangers. In particular we hypothesized that (1) dyads of couples receiving the micro-intervention would choose more prosocial outcomes in a behavioral game than dyads of couples that did not receive the intervention; (2) that dyads of couples in which only one partner received the micro-intervention would show less prosocial outcomes than dyads of couples in which both received the intervention but more than dyads in which neither received the micro-intervention; and (3) that individuals who received the intervention would show increases in prosocial behaviors across the week, as measured in an ESM procedure.

We first examined if the micro-intervention designed to increase the psychological flexibility of participants would lead to more giving behavior in the Dictator Game^20^. The Dictator Game is a widely used behavioral game that asks participants to decide how much of a fixed amount of money should be given to their partner and how much they would keep for themselves. In this study, participants decided how much of their participant compensation (240 Francs; 1 Franc equals approximately 1 USD) would be split into gift certificates to real stores that they liked, but their partner did not and vice versa. The rating of the stores was previously undertaken by each participant separately. Using mutually exclusive gift certificates prevented them from choosing something to simply share with their partner later. After the completion of the study (i.e., subsequent to the behavioral game), participants were compensated up to 240 Franks for the time they invested in the study irrespective of their actual responses to the Dictator Game.

## Results

In the first preliminary study, the dyads of couples were randomized to either receive the micro-intervention (n = 10) or not (n = 6). All dyads completed the Dictator Game in the laboratory one week later. Participants who received the micro-intervention training psychological flexibility allocated on average 146 Francs (SD = 49.9) to their partners in form of gift cards for shops their partner liked but they did not, whereas those who did not receive the micro-intervention allocated on average only 120 Francs (SD = 0.0). The allocation was 21.6% higher, d = 0.74.

Having observed the increase of amount of money given to the partner due to the micro-intervention one week later, we replicated this preliminary study with a larger sample and additional control groups to isolate the mechanisms of action and to rule out some threats to internal validity. Thus, in Study 2 we randomized dyads of couples now to one of four groups: both partners received the micro-intervention and ESM with exercises and questions about altruistic behavior (Group 1); only one partner received the micro-intervention, but both received the ESM (Group 2); neither received the micro-intervention, but both received the ESM (Group 3); and neither received the micro-intervention nor the ESM (Group 4). The introduction of Group 2 allowed us to test the dose–response of the micro-intervention (i.e., do both partners in a dyad need to receive the intervention for the altruistic behavior effect to emerge or is one enough?) and the introduction of Group 4 allowed us to control for the effect of the ESM.

**Figure 1:**
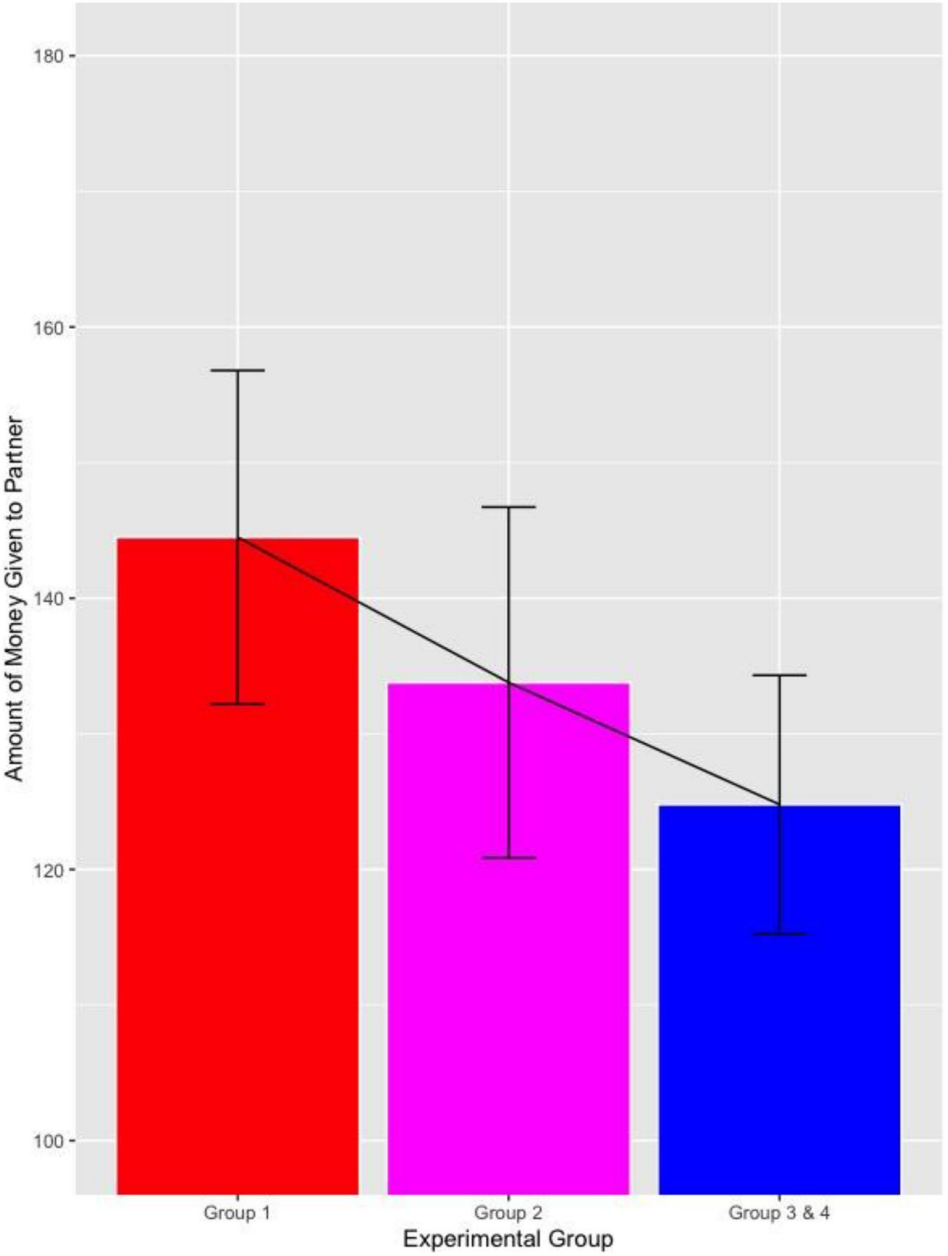
Mean value and standard error participants gave to their partners, by group.

**Figure 2:**
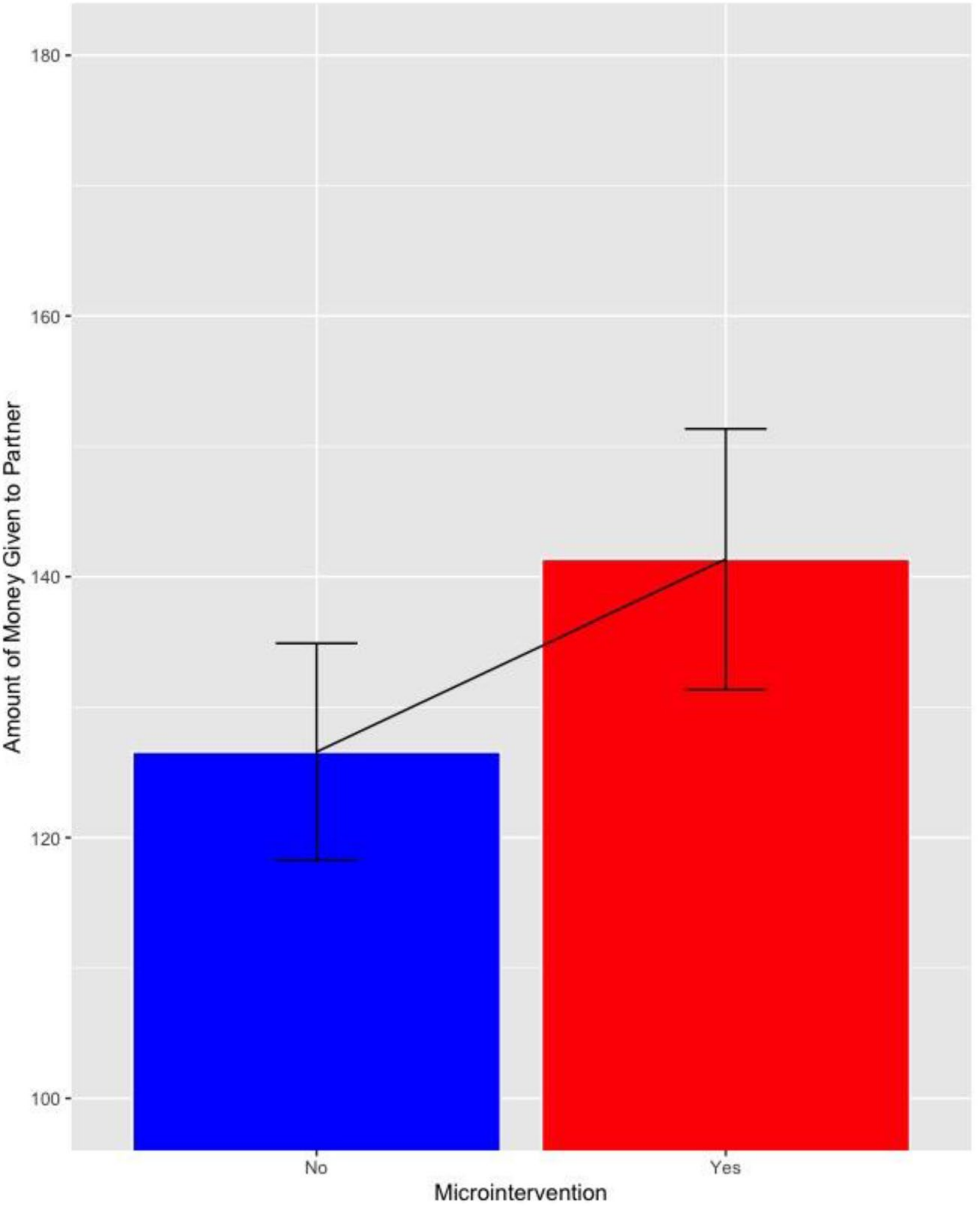
Mean value and standard error participants gave to their partners, by those that did and did not receive the micro-intervention.

In this study, all participants were tested on the standardized Dictator Game one week after receiving the micro-intervention. Once again, we saw that participants that received the micro-intervention engaged in more prosocial behavior, with evidence of a dose–response effect (i.e., dyads of couples where both participants received the micro-intervention tended to respond more prosocially than dyads of couples where only one partner received the micro-intervention). The mean amount participants gave their partner in each group was: Group 1 (Francs 144.5, SE = 6.2); Group 2 (Francs 133.8, SE = 6.5); Group 3 (Francs 124.0, SE = 6.72); and Group 4 (Francs 125.6, SE = 6.99). Because Group 3 and 4 differed only with respect to ESM and there was no statistical difference between them, we combined these groups for subsequent analyses. The amount participants gave to their partner was highest when both partners received the micro-intervention (Group 1) and this amount decreased linearly across the groups (p = 0.014, for linear polynomial across the three Groups 1, 2 and 3 & 4 combined, using a multilevel model with participants as lower and dyad as upper level, including a random intercept; see Fig. 1; (for group comparisons see supplementary material). Given that half the participants in Group 2 received the micro-intervention and half did not, we split these participants into respective groups and examined the effect of all those who received (n = 87) vs. did not receive (n = 127) the micro-intervention. This effect was even stronger, with those receiving the micro-intervention giving on average Francs 141.3 (SE = 5.0) whereas those that didn’t receive the micro-intervention gave 126.6 (SE = 4.2; d = 0.32, p = 0.024 for difference between the two groups, based on multilevel model (see Fig. 2).

**Figure 3:**
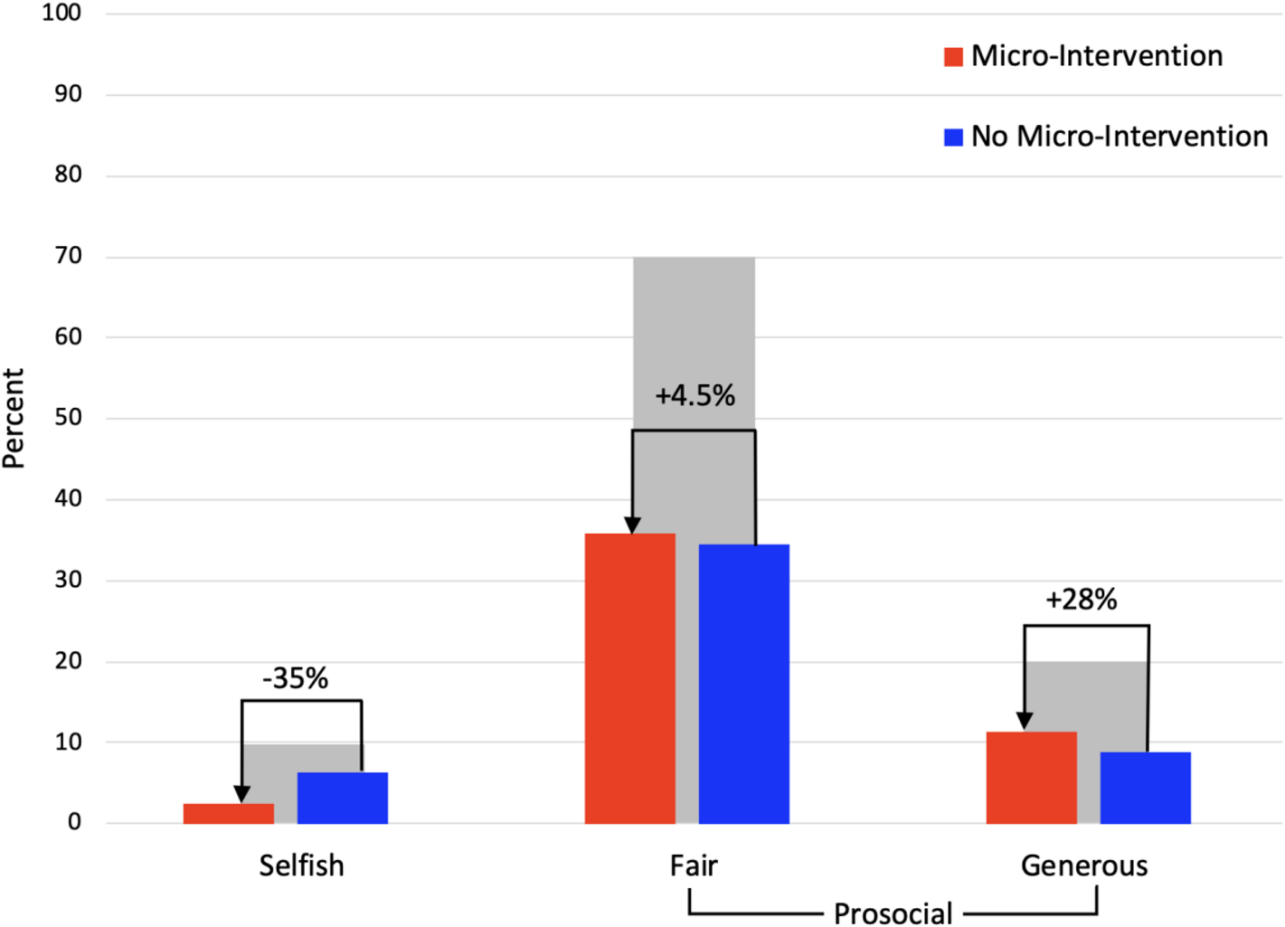
Percentage of participants who responded selfishly, fair, and altruistically across all groups (grey bars). Each response category is further split into those that received (red bars) vs did not receive the micro-intervention (blue bars) and the percent difference between these groups.

Participants were free to share any amount of their endowment (i.e., 0–240). In so doing, the Dictator Game forces participants to decide if they will divide the money prosocially (i.e., fairly (50/50) or giving more to their partner (generous)), or keep more for themselves (selfish). In order to further understand how these dimensions impacted responding, we grouped participants’ responses accordingly. Across all four groups, 70.1% of participants responded fairly (50/50), 20.1% generously, and only 9.8% selfishly. Within groups, more prosocial responses (fair and generous divisions) were recorded in Groups 1 and 2 and three times more selfish responses were recorded in Groups 3 and 4. In order to further specify the impact of the intervention, we once again split participants into those that received the micro-intervention (n = 87) and those that did not (n = 127). In participants who received the micro-intervention, 72% responded fairly, 23% generously, and only 5% selfishly. In comparison, those that did not receive the micro-intervention 69% responded fairly, 18% generously, and 13% selfishly. Importantly, of those that responded selfishly, 81% (17 out of 21) did not receive the micro-intervention vs. only 19% (4 out of 21) in those who did (see Fig. 3).

**Figure 4:**
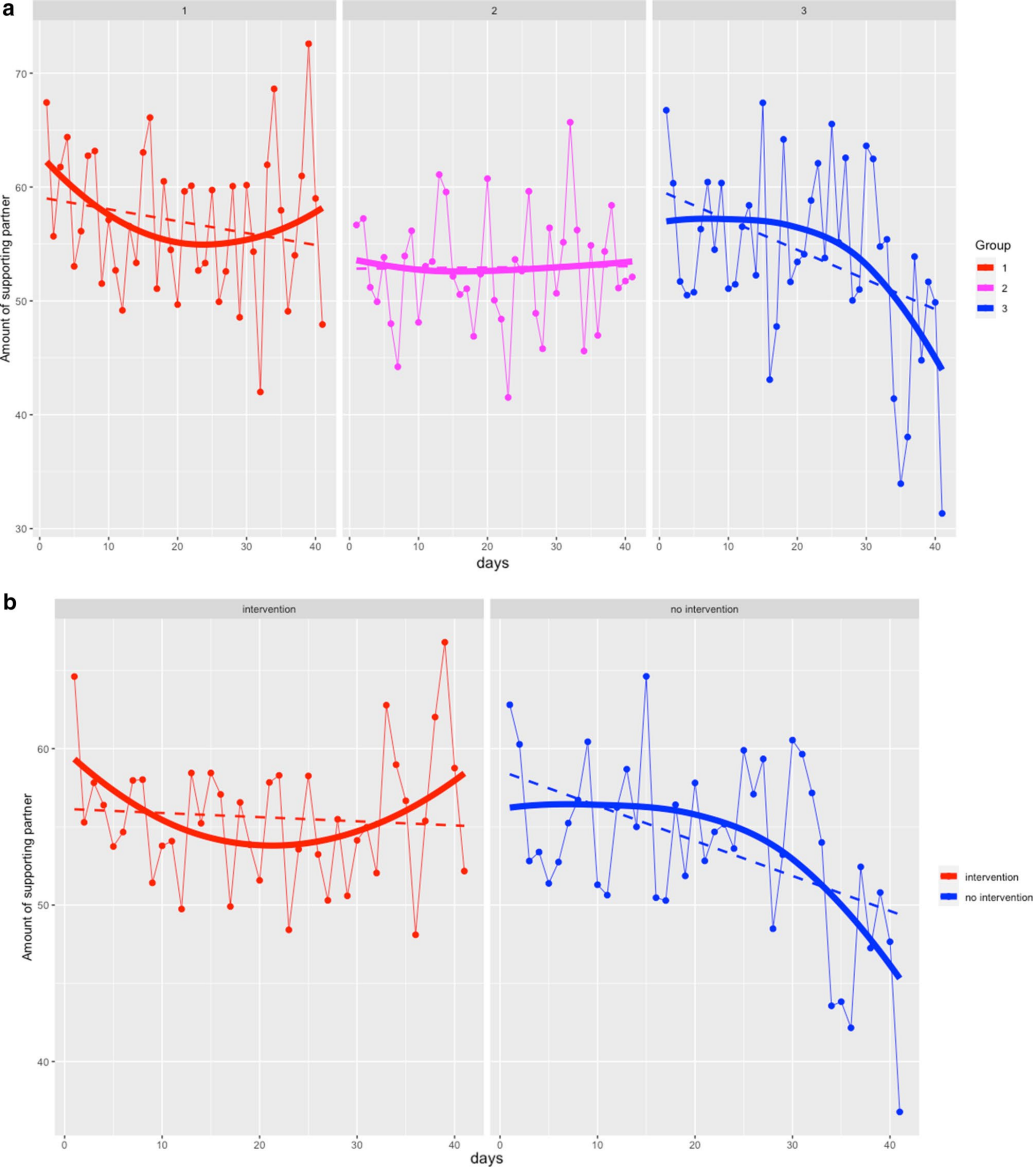
Amount of support given to partner across the week as measured using ESM, by group (**a**) and those that received vs. did not receive the intervention (**b**).

Despite the pattern of results observed in the laboratory, it cannot be assumed that this generalizes to participants’ behavior in their everyday lives. We therefore prompted participants in groups 1–3 five times per day and queried about their prosocial behaviors in the form of whether they spontaneously supported their partner. Figure 4a shows the patterns across the three groups. Although Group 3 exhibited a decline that became stronger and stronger over the study period, there was no interaction effect of group x days (neither linear nor quadratic). When the participants were split into micro-intervention vs. no micro-intervention, however, participants who received the micro-intervention exhibited a different trajectory over the study period than those without. Those with the micro-intervention first slightly declined but then increased their prosocial behaviors again towards the study end, whereas participants without the micro-intervention declined more and more towards the study end. The interaction effect of group × days for a linear trend was not significant (p = 0.167), but was for a quadratic trend (interaction quadratic polynomial for time x intervention on partner level, p = 0.014; see Fig. 4b).

## Discussion

This study found that a micro-intervention targeting psychological flexibility increased prosocial behavior and decreased selfish behaviors in dyads made of couples in relationships. Importantly, these results were observed in both the laboratory and participants’ self-chosen environments from their everyday lives (ESM). Results suggest a dose–response pattern such that more altruistic behaviors were observed in groups where both partners received the micro-intervention.

A meta-analysis of Dictator Game studies using strangers found that, on average, participants give 28.35% of their share to their partners. A third of the participants keep everything, and 17% split their amount 50/50 with the recipient^4^. The present findings, with approximately 70% of the sample splitting the money 50/50 and 20% giving more to their partner, points to a pointedly different distribution. As observed here with dyads of couples in a relationship, prosocial distributions are much more common than with strangers. By using dyads of couples in real relationships (instead of strangers) it is likely that trust and concerns of being exploited—factors found to impact prosocial choices^21,22^—by the other person differed from studies conducted with strangers. It is likely that couples in real relationships may be influenced by the desire to share or express affection. Indeed, just the expectation of having future interactions with someone has led to an increase in altruistic behavior in self-protecting individuals^23^. Given that couples are the most prevalent social group among humans, these findings suggest that future studies should also examine this and other naturally occurring groups. Importantly, we observed an increase in prosocial behavior among participants who received the micro-intervention above and beyond the already greater prosocial responses on average in this study relative to strangers.

Some studies suggest that prosocial behaviors can be increased via contextual and procedural variations such as forming social norms^24^, being observed by your child^25^, or even simply seeing someone’s name^26^. However, few studies have attempted to actively intervene to increase prosocial behaviors with skills under the voluntary control of participants. One such study found that mindfulness training (eight weeks) led to a trend in increasing prosocial orientation as assessed via questionnaire^27^. Similarly, two weeks of compassion training led to participants choosing more prosocial options towards a victim than participants in a control condition after witnessing an unfair encounter^28^. The present study highlights that a very brief intervention aimed directly at the mechanism of psychological flexibility—which is known to increase awareness of values such as caring and supporting a partner while also removing barriers from acting on those values—led to increased prosocial behavior as observed in the behavioral games and in their daily lives.

This study also highlights the possibility and importance of using a multi-method approach to the topic of prosocial behaviors. Previous studies concentrated on retrospective recalled questionnaires, laboratory games, or observances in the field. The approach chosen in this study combined these and thus had the advantage of cross-verifying observations across multiple time points and contexts.

It is well known that social interactions are crucial for people’s well-being and it has been found that people who are more prosocial have better relationships^29,30^. Likewise it is well known that people are more prosocial to people they are close with compared to distant people^31^. It has also been found, however, that overly self-sacrificial individuals may be prone for depression—at least when measured among participants who were strangers^32^. To the degree that increasing prosocial behaviors and decreasing selfish behaviors can thus decrease loneliness and social discord, such interventions could prove relevant for multiple contexts. This study demonstrated the impact of a micro-intervention. This is important because in order to be scalable, any such intervention must also be short and easy to implement.

This study was limited in at least two ways. First, although participants were playing for real money in the form of mutually exclusive gift certificates, we cannot rule out that some participants made decisions during the Dictator Game with the intent to share the money. We did not, however, find evidence of this and if it occurred, then it was random. Second, ESM asks participants to reflect on their own experiences and thus is subject to some of the biases of self-report. That said, ESM allows for assessing behavior in everyday life in participants naturally occurring daily environments and routines as chosen by the participants; ESM therefore has high ecological validity and limits recall bias^33^.

These limitations notwithstanding, this study demonstrated across multiple methods in the laboratory and in everyday life that a micro-intervention increased prosocial behaviors and reduced selfishness. The effects of the micro-intervention were strongest in dyads where both participants received the intervention. These findings are relevant for researchers interested in prosocial behaviors, altruism, and selfishness as well as dyads. This study contributes to the growing stream of papers exploring how families impact prosocial behaviors^25^ and future research should study these relations in addition to studies on strangers. Given the brevity of the micro-intervention and that it trains skills under voluntary control of participants, it has the potential to be implemented in numerous contexts and contribute to prosocial change in social interactions.

## Materials and methods

### Data reporting

Results from the preliminary study were used to calculate expected effect sizes for the main study. Both studies were randomized, with participants blinded to the allocated treatment, and investigators blinded to the study’s hypotheses.

### Ethics approval and consent to participate

This study was approved by the local ethics committee (University of Basel, Department of Psychology Ethics Committee IRB Nr. 001-15-2). All experiments were performed in accordance with the ethics committee guidelines. All subject signed an informed consent before participation.

### Subjects

In the first preliminary study, n = 8 couples (n = 16 individuals; 8 women and 8 men) were recruited from local advertisements. In the second (main) study, 494 individuals we recruited from local advertisements. In total 126 couples (252 individuals) were randomized to one of four study groups and 118 couples (236 individuals) entered pre-assessment. Five couples (10 individuals) were lost to dropout from T1 to T2 assessment. Missing data from single participants resulted in the exclusion of two dyads (four individuals) resulting in a final sample size of n = 111 dyads of couples (222 individuals). The sample consisted of 114 females and 108 males, with most couples reporting a heterosexual relationship. Participants were on average 32 years old (SD = 12.24), range 18–75 years, with an average length of relationship of 7.8 years (SD = 9.04), range 0.5–44 years. The groups were balanced for age and length of relationship and did not differ significantly. Participants were very compliant with the ESM assessments: 96.4% of the individuals filled out more than 50% of the smartphone prompts. For the analyses involving ESM, 6 individuals were excluded from the analysis because they filled out less than 50% of the smartphone prompts.

### Randomization

Dyads of couples were randomized into one of four groups. Random blocks were generated using computer-generated sequences. Two strata were created: one for couples over 30, and one for couples under 30 years old. An administrator not involved with data collection communicated the group assignment.

Couples were randomized before the T1-assessment to one of four study groups. In Group 1 both individuals of a couple additionally received a brief psychological flexibility intervention and were given a paper pencil diary that they could use to further practice aspects of psychological flexibility. In Group 2 only one individual of the couple, received the micro-intervention and the diary, and in Group 3 neither of the two individuals received the micro-intervention or the diary. All individuals in Groups 1–3 received the ESM to complete between T1 and T2. Finally, in Group 4, individuals neither received the micro-intervention, nor the diary, nor the ESM assessment. For the couples in Group 2, an additional randomization determined which of the partners would receive the micro-intervention and which would not.

### Study procedure

Within the first participant contact (E-mail or phone) we checked whether potential participants met study criteria. To be eligible to take part in the study participants had to be at least 18 years old, currently in a relationship for at least 6 months, in daily contact with their respective partners, able to attend together as a couple two appointments at the university site, did not have hearing difficulties and were not red-green blind. If the study criteria were met, we then invited eligible participants to two appointments (T1 and T2) to the local university site, which had to be 7 days apart. At T1, participants completed questionnaire packets (data not presented here). Between T1 and T2, participants in Groups 1–3 engaged in ESM and completed questions on a smartphone device six times a day (e.g., 7:00/10:00/13:00/16:00/19:00/22:00). At T2 (a week after T1) all participants once again completed questionnaires and engaged in the behavioral tests.

### Prosocial behavior

We operationalized prosocial behavior using the Dictator Game, and specifically developed prosocial behavior items presented repeatedly on the smartphone device.

The Dictator Game is a well-known behavioral test which traditionally assesses altruistic behavior between two strangers. The game asks a player to decide how much money he or she wants to give to the other player^20^. Both individuals in the dyad were instructed to split 240 Francs worth of gift certificates between them and their respective partner.

To further measure altruistic behavior in a daily social context we asked participants two questions on the smartphone five times a day for a week (not during the first morning prompt). First, we asked, if they “yes” or “no” helped someone (“Since the last prompt, have you either helped someone, took care of someone, supported someone, or did something for someone else?”). Second, they were asked if they supported their partner (“since the last prompt, how much did you support your partner?”). Support is measured on a likert scale from “0” not at all to “100” very much.

### Micro-intervention

The micro-intervention trained skills previously shown to promote psychological flexibility^34^. The micro-intervention took on average 15 min. In a first step, participants were instructed to describe struggles and stress they are currently experiencing within their daily life. In a second, they were asked about people, activities, and other things in their lives that are genuinely important to them (i.e., values). Using an experiential metaphorical exercise, participants were asked to experience how their struggles interfered with the things they valued. Then, skills of present moment awareness and acceptance were practiced with the goal of experiencing more flexibility to make room for the things with which they struggle. Thereafter, the relation to their values was once again probed. The overarching goal of the short exercise was to experience that pushing away, avoiding, and struggling with stress makes it more difficult to interact with meaningful individuals and do meaningful activities. The introduced skills aimed to help participants be aware of this, choose whether to use acceptance, and to consciously focus on meaningful people and activities.

This flexibility training was written down including practice instructions within a dairy that was given between T1 and T2. Participants were instructed to practice the exercises once a day. The skills included in the diary were the same as in the exercise and included present moment awareness (via an audio file), thinking about their personal values, acceptance, contemplating how to engage more with their values, as well as reflecting about barriers to implementing valued orientated behavior.

### Statistical analyses

To analyze the data of the Dictator Game we used a multilevel model with amount of money allocated to partner as outcome, dyad and partner (within dyads) as level 2 and 1 variables, respectively, and either intervention on dyad level (both partners/one partner/none receiving micro-intervention) or intervention on partner level (intervention/no intervention) as between-subjects factor.

Data on the temporal trajectories of prosocial behavior of partners and of psychological flexibility across one week were analyzed using a three-level model, with either amount of supporting partner or individual psychological flexibility as outcome, dyad, partner (within dyads), and time (within partners) as level 3, 2, and 1 variables, respectively, and intervention on partner level (intervention/no intervention) as between-subjects factor.

## Supplementary Information

Supplementary Information.

## Data Availability

Aggregated data used in this study is available on the open science framework (OSF). https://doi.org/10.17605/OSF.IO/7WFJC.
