# Supplementary material for: Increasing prosocial behavior and decreasing selfishness in the lab and everyday life
Source: Sci Rep. 2020 Dec 4;10:21220. doi: 10.1038/s41598-020-78251-z (PMC7718232; doi:10.1038/s41598-020-78251-z)
Supplement: Supplementary file 1 — Supplementary Information. [file 41598_2020_78251_MOESM1_ESM.docx]

**Increasing Prosocial Behavior and Decreasing Selfishness in the Lab and Everyday Life**

Andrew T. Gloster^1^*, Marcia Rinner^1^, Andrea Meyer^2^

^1^University of Basel, Faculty of Psychology, Division of Clinical Psychology & Intervention Science

^2^University of Basel, Faculty of Psychology, Division of Clinical Psychology & Epidemiology

*Corresponding to:

Prof. Dr. Andrew T. Gloster

University of Basel

Department of Psychology

Division of Clinical Psychology and Intervention Science

Missionsstrasse 62 A

4055 Basel

Switzerland

E-mail address: [andrew.gloster@unibas.ch](mailto:andrew.gloster@unibas.ch)

Phone number: +41 61 207 02 75

Supplemental Material

Pairwise comparisons between the groups were conducted between groups 1, 2, and 3 & 4, as follows. Only the contrast between groups 1 and 3+4 was significantly different.

| Contrast | Estimate | SE | *df* | *t* | *p* |
| --- | --- | --- | --- | --- | --- |
| Group 1 vs 2 | 10.71 | 9.00 | 105 | 1.19 | 0.462 |
| Group 1 vs 3+4 | 19.71 | 7.84 | 105 | 2.51 | 0.036 |
| Group 2 vs 3+4 | 9.00 | 8.10 | 105 | 1.11 | 0.509 |
